# Trichodiene: a committed intermediate and a chink in the armor of mycotoxigenic *Fusarium*species

**DOI:** 10.1128/aem.00150-26

**Published:** 2026-05-26

**Authors:** Subbaiah Chalivendra

**Affiliations:** 1 Anschutz Medical Campus, University of Colorado 129263, Aurora, Colorado, USA; Michigan State University, East Lansing, Michigan, USA

**Keywords:** trichodiene, xanthotoxin, biofumigation, mode of action, ergosterol, trichothecenes, substrate inhibition

## Abstract

Trichothecenes, mycotoxins produced by some *Fusarium* species, threaten crop production, animal health, and food safety. Among them, deoxynivalenol (or vomitoxin) is a major contaminant of cereals targeted by resistance breeding and chemical or biological controls. Trichodiene, the volatile intermediate in the trichothecene biosynthesis pathway, reduces mycotoxin production and shows promise as a source of biocontrol. A pilot-scale fungal fermentation and purification method (W. T. Hay, N. D. Kemp, A. R. Payne, N. Rhoades,et al., Appl Environ Microbiol92:e01695-25, 2026,https://doi.org/10.1128/aem.01695-25)now enables *in planta* testing for its potential in suppressing fungal colonization and toxin accumulation in cereals.

## COMMENTARY

**Fig 1:**
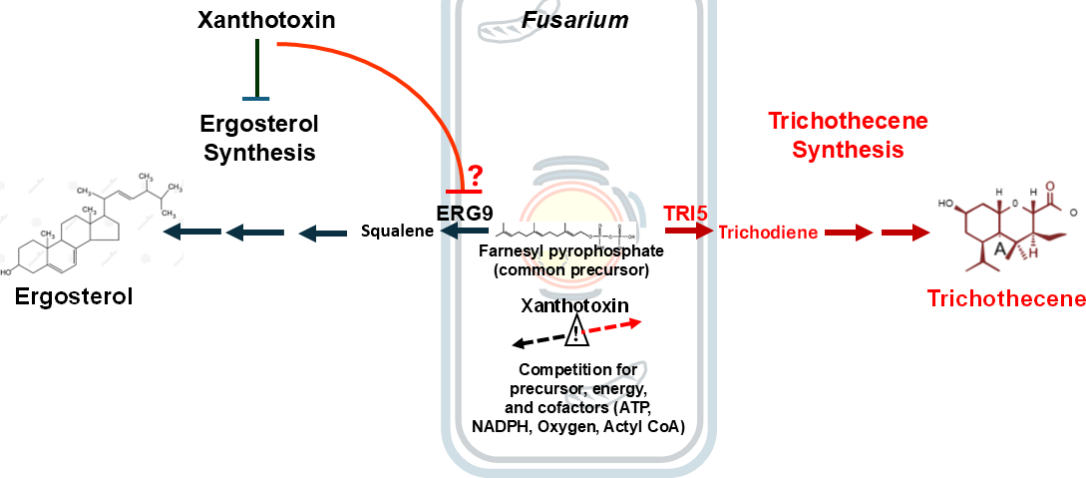
Schematic showing the proposed mode of action of xanthotoxin. Xanthotoxin seems to increase trichodiene synthesis by inhibiting the incorporation of FPP into squalene, the first step of ergosterol biosynthesis. Thereby, the furanocoumarin increases the flow of the shared precursor into trichothecene biosynthesis at the expense of ergosterol biosynthesis.

*Fusarium* species cause not only economically devastating diseases in globally important crops (e.g., wheat, maize, and potato) but also contaminate food and feed with mycotoxins, threatening human and livestock health (1, 2). They produce a diverse array of mycotoxins, of which the most notable are trichothecenes, such as deoxynivalenol (DON or vomitoxin), T-2 toxin, and sesquiterpenoids, all made using farnesyl pyrophosphate (FPP) as the precursor. This class of mycotoxins is primarily produced by fungal pathogens of cereal crops, causing Fusarium head blight (FHB). *Fusarium graminearum and Fusarium culmorum* colonize wheat and barley, whereas *Fusarium langsethiae* and *Fusarium sporotrichioides* often cause FHB in small-grain cereals such as oats (3). There has been measurable success in wheat resistance breeding for FHB. New resistant cultivars are currently in the final stages of testing (4). Biocontrol agents have also been tested for FHB in wheat but are found to be less efficacious than conventional agrochemicals (5). Azole fungicides, specifically triazoles, are the most effective in controlling FHB ([6] and references therein). However, heavy pesticide pressure can lead to resistance development to these chemicals in *F. graminearum* (reviewed in reference 7). Furthermore, many azole fungicides increase trichothecene levels if applied at sublethal levels (8; reviewed in reference 9). This is because azole fungicides target the ergosterol synthesis pathway, which also uses FPP as the precursor (10). Inhibiting ergosterol biosynthesis enhances trichothecene biosynthesis due to an increased availability of the precursor, ATP, and other cofactors common to both pathways (Fig. 1). Therefore, additional and complementary control measures against trichothecene contamination of the food chain need to be developed as part of integrated pathogen management. It is preferable that such strategies are broadly effective against diverse *Fusarium* species and crop hosts, in addition to wheat (which surely is more widely grown as a staple food crop).

Trichodiene fumigation represents a promising alternative strategy for trichothecene control, although additional work is needed to address outstanding field deployment challenges. The following section summarizes key observations that led to the discovery of trichodiene as a potential strategy for trichothecene mitigation. Xanthotoxin, a plant secondary metabolite and known inhibitor of P450 oxygenases, was first found to block the synthesis of the T-2 toxin in *F. sporotrichioides* (11, 12). Furthermore, the study showed that the inhibition is accompanied by the stimulation of trichodiene, the pathway-specific precursor of trichothecenes. Furthermore, xanthotoxin increased trichodiene levels even in the TRI4 mutant (which is deficient in the P450 enzyme that oxygenates trichodiene), ruling out P450 oxygenase inhibition as the mode of action. Building on an earlier report in tomato (13), Taylor et al. (14) tested whether trichodiene acts as a signaling molecule in its inhibition of trichothecene production and if an external application of trichodiene can control mycotoxin synthesis *in planta*. Wheat plants pre-inoculated with trichodiene-producing *Trichoderma* (Th+TRI5) accumulated less DON and exhibited reduced disease severity following infection with *F. graminearum* compared to plants treated with unmodified *Trichoderma* or untreated controls (13). Th+TRI5 volatiles also reduced DON in *F. graminearum*. However, the volatiles had no effect on fungal growth in culture. Fumigation with purified trichodiene also reduced DON content in excised wheat heads of two cultivars but made no differencein disease intensity (13).

In the most recent study (15), the authors developed a pilot-scale fermentation method to bulk produce trichodiene using a *TRI4* mutant strain of *F. sporotrichioides* and xanthotoxin treatment to enhance the yield. The fermentation conditions, such as xanthotoxin concentration, fermentation time, and extraction solvent, were optimized using a benchtop scale fermenter. Purified trichodiene showed a consistent reduction of mycotoxin either in contact or fumigation assays but was highly variable in its effect on fungal growth (15). In order to realize its promising effect on mycotoxin reduction, it is imperative to demonstrate the ability of trichodiene to curb fungal growth. There are additional challenges associated with its volatility and potential biodegradability. Controlling mycotoxigenic fungal diseases poses added complexity, since host colonization can proceed before mycotoxin production. This is particularly conspicuous in the case of fungi, e.g.,*Aspergillus flavus*, whose major mycotoxins, i.e., aflatoxins, have no or minimal phytotoxicity and are not required for infecting the host. In *A. flavus*, cyclopiazonic acid (another toxin made by the fungus) is a key pathogenicity factor (16). However, trichothecenes are phytotoxic and have been shown to be important in host colonization using knockout mutants in the trichothecene biosynthetic pathway (17, 18), implying the need for further optimization of trichodiene treatment to adequately reduce trichothecene levels and prevent colonization. A proven alternative to pure trichodiene fumigation may be the biocontrol fungus, *Trichoderma* engineered to overexpress terpene cyclase (TRI5) under a strong promoter (e.g.,reference 14). To avoid problems observed in liquid fermentation of *Trichoderma* (15), solid-state fermentation routinely used in the bulk production of fungi can be adapted (19).

It is also important to fully probe the mode of action of xanthotoxin, not only in the interest of our understanding but also from the disease control perspective. The ability of xanthotoxin to increase trichodiene even in the *tri4* mutant (12) suggests that it acts by an increased channeling of FPP into trichodiene synthesis. The other major and constitutive pathway that utilizes FPP as the precursor is ergosterol biosynthesis. It follows that xanthotoxin may act as an inhibitor of ergosterol biosynthesis in its promotion of trichodiene accumulation (Fig. 1). A decrease in wild-type *F. sporotrichioides* growth after a 24-h exposure and a drastic decrease in tubulin mRNA within an hour of exposure to xanthotoxin (12; also see reference 20) also suggest the repression of ergosterol synthesis as xanthotoxin’s mode of action. A recent report confirms that the repression of fungal growth by xanthotoxin is associated with the inhibition of ergosterol production (21). One critical constraint to this hypothesis is that xanthotoxin should act at the first step of ergosterol biosynthesis, i.e., by inhibiting squalene synthase (ERG9) and allowing an increased flow of FPP into trichodiene biosynthesis (Fig. 1). Otherwise, the hypothesis will be nullified. As alluded to above, azole fungicides are known to enhance trichothecene biosynthesis (8). They act by inhibiting lanosterol 14α-demethylase (ERG11; i.e.,two steps downstream to FPP conversion to squalene). The *tri4* mutant, in contrast to the wild type, seems to maintain some level of ergosterol biosynthesis, as accumulated trichodiene might lead to product inhibition of FPP cyclase (TRI5). This prediction is consistent with the lower sensitivity of the *tri4* mutant to xanthotoxin-imposed growth inhibition than the wild type (12). It is worth testing non-phytotoxic fungicides/chemicals that can inhibit ergosterol at the ERG9 step on trichodiene and trichothecene biosynthesis.There are not many such inhibitors. Terbinafine, although an inhibitor of squalene oxidase (ERG1), is known to affect squalene synthase activity, presumably by product inhibition (22). Squalestatin 1 (a pharmaceutical, like terbinafine), a potent inhibitor of squalene synthase, may enhance FPP flow into trichodiene biosynthesis (23).

**Fig 2:**
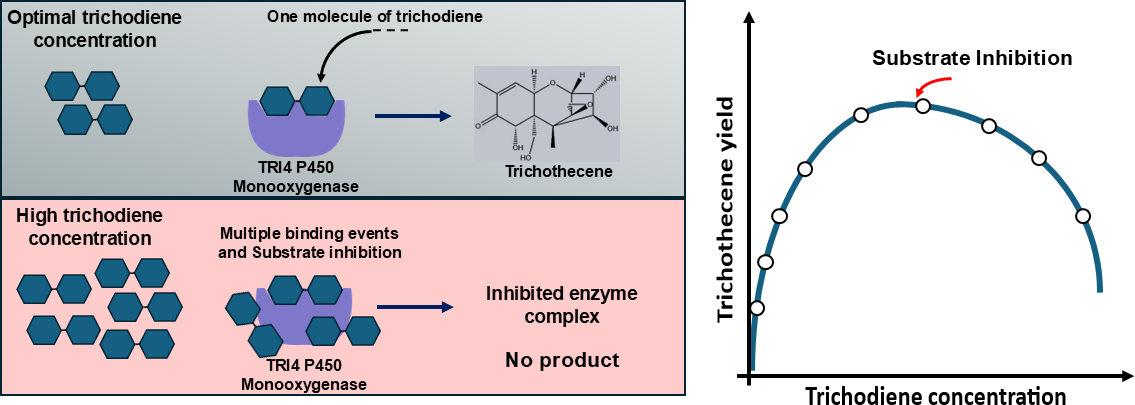
Inhibition of TRI4 cytochrome P450 enzyme by supra-optimal levels of trichodiene may be the underlying mechanism of trichodiene’s effect on trichothecene biosynthesis. Substrate inhibition is not uncommon for cytochrome P450 enzymes (25, 26).

Another important question yet to be addressed is how an increase in trichodiene, a committed intermediate of the biosynthetic pathway, leads to the inhibition of trichothecene production. It is not surprising that trichodiene accumulates in the *tri4* mutant, particularly when treated with xanthotoxin, as the sesquiterpene intermediate cannot be processed further into trichothecene biosynthesis. However, this rationale does not apply to the wild type. Why does an external addition of trichodiene or its endogenous increase by xanthotoxin inhibit trichothecene biosynthesis in the wild -type strain despite having fully functional TRI4 activity? Here is one possible explanation. The inhibitory effect of excess trichodiene on trichothecene synthesis may be due to substrate inhibition of TRI4 cytochrome P450 enzyme (Fig. 2). Substrate inhibition is the most common deviation from Michaelis-Menten kinetics. It occurs in nearly 25% of enzymes due to allosteric effect of two or more substrate molecules binding to the enzyme or due to the blocking of product release from the enzyme-product complex (24). It is even more common in cytochrome P450 enzymes, up to 50% of all studied enzymes (25, 26). The substrate inhibition of TRI4 and the accumulation of trichodiene (Fig. 2) seems to cause a feedback inhibition of pathway (*Tri*) gene expression, including those coding for isotrichodiol synthase, the first cytochrome P450 that metabolizes trichodiene (TRI4), and one of the transcription factors (TRI6) that is required for the expression of *Tri* genes (12, 14, 18). Enzyme kinetics assays with purified TRI4 P450 oxygenase could easily test substrate inhibition as well as any potential inhibitory effect of xanthotoxin on TRI4. The scaled-up production of trichodiene reported by Hay et al. (15) opens opportunities to test this and other hypotheses and gain mechanistic insights into its inhibitory effects. The research group needs to be commended for taking their work beyond academic interest.
